# Supplementary material for: Connecting Diagnostics and Clinical Relevance of the α-Gal Syndrome—Individual Sensitization Patterns of Patients with Suspected α-Gal-Associated Allergy
Source: Nutrients. 2025 Apr 30;17(9):1541. doi: 10.3390/nu17091541 (PMC12073179; doi:10.3390/nu17091541)
Supplement: Supplementary file 1 [file nutrients-17-01541-s001.zip › nutrients-3582741-supplementary.pdf]

Supplemental Information

Table S1. Individual clinical history of patients with suspected AGS. Grey background indicates patients with negative serum test for IgE against Bos d TG (α-Gal analyte) in ImmunoCAP (see Table 1). AD, atopic dermatitis; EoE, eosinophilic esophagitis; n.a., not applicable; GI, gastro-intestinal; HDM, house dust mite; OAS, oral allergy syndrome; OC, oral challenge; SPT, skin prick test.

ID	Age	Sex	Allergy History	Symptoms after α-Gal-ingestion	Reaction Interval	Co-factors	Report on tick bite	Clinical reaction to tick bite	Symptoms after milk consump-tion	Symptoms after gelatin consump-tion	OC-Results	Additional Information
**P01**	17	m	Idiopathic anaphylaxis	Red meat, cheese: Urticaria; pruritus; angioedema; erythema; gastrointestinal cramps; diarrhoea; tachycardia.	2-3 hrs	Exercise	No	n.a.	No	No	OC including co-factors was negative for meat, kidney, gelatin	Vegetarian, suspected allergy to rennet in cheese
**P02**	30	m	HDM allergy in childhood; Now inhalant allergy to tree pollen; recurrent angioedema of lips and tongue	Recurrent angioedema of lips and tongue with slow resolution. Crescendo of the symptoms: dyspnea, abdominal cramps, massive diarrhea; angioedema to gelatin in drugs against a bronchial infection. Dose dependency of symptoms.	8-12 hrs; nocturnal angioedema	Infection	Yes	prolonged erythema	No	Yes; Anaphylaxis to gelatin in drug	n.a.	Professional cook [30]
**P03**	32	m	Inhalant allergy to timothy grass and herbal pollen	Pruritus arms, head, ears, erythematous reaction, loss of consciousness, diarrhea, dyspnea	15 min to 3 hrs	None	No	n.a.	No	No	n.a.	n.d.

**P04**	29	m	Inhalant allergy to horse, dog, HDM in childhood that has resolved, no AD	Pruritus head, whole body, urticaria, angioedema, dyspnea, tachycardia; nausea, drop of blood pressure; dysphagia	Cannot be specified	Stress, alcohol	Yes	none	No; Tolerance instead	No	None performed	Tick bites in childhood. Tolerance to milk and muscle meat.
**P05**	23	f	AD in childhood. Inhalant allergy to grass pollen, HDM, with asthma. Peanut, nut, wine gum, kiwi (OAS)	Anaphylaxis grade II, dyspnea, urticaria, eyes, nose, laryngeal oedema, gastrointestinal symptoms with flatulence and loss of appetite	3 hrs after wine gum consumption	None	No	n.a.	No	Yes	OC showed tolerance of bovine muscle meat; however, kidneys were not used for challenge	No additional information
**P06**	55	m	No atopy. Hymenoptera venom allergy	Urticaria, pruritus, collapse	6 hrs	Stress, alcohol	Yes, 40/y	none	No	No	OC with milk and gelatin: negative	Professional cook, hunter
**P07**	69	m	HDM allergy	Swelling of the tongue, cardiovascular reactions	30 min-2.5 hrs; sometimes nocturnal angioedema of the tongue	Stress; once an infection as a probable co-factor	Yes	none	No	No	n.a.	Tick bites 10 yrs. before
**P08**	78	m	Sensitization to inhalant allergens (tree pollen and HDM); Milk allergy (self-provocation)	Urticaria, pruritus	20 min (milk) 80 min (pork kidney)	None	Yes	none	Anaphylaxis	No	OC positive for milk and pork kidney, negative for pork meat and gelatin	Tick bite in 2020 [29]

**P09**	55	m	No atopy	Recurrent angioedema and urticaria, sometimes swelling of the tongue;	Up to 12 hrs	none	No	n.a.	No;<br><br>Tolerance instead	No	n.a.	Symptoms occur also after eating other food, poultry
**P10**	56	f	Multiple contact allergies; no atopy.	Angioedema (face, genitalia), localized swellings.	Several hours	none	No	n.a.	No	No	n.a.	No tick bites remembered, but slow-healing small wound on her right leg.
**P11**	55	f	Inhalant allergy (birch) and pollen-associated food allergy	Anaphylaxis (sausage with curry): tremor, nausea; swelling of the tongue; tightness of the throat; sweating, dizziness. Months later: Nocturnal angioedema	Several hours	Alcohol	Yes	with pruritus	No;<br><br>Tolerance instead	No;<br><br>BUT: SPT gelafundin >6mm (+)	n.a.	Avoidance of beef but consumption of pork.
**P12**	20	m	Sensitization to several plant foods and *Crustazeae*	Idiopathic anaphylaxis, exanthema, pruritus; angioedema; once loss of consciousness; dyspnea;	Nocturnal anaphylaxis, not always meat consumption remembered	Exercise	Yes	none	No	No	n.a.	No additional information
**P13**	32	f	Inhalant allergy to tree pollen and HDM; pollen-associated food allergy and asthma; contact urticaria to raw potato; recurrent anaphylaxis to foods (idiopathic anaphylaxis)	Recurrent anaphylaxis, OAS, rhinitis; dyspnea, tachycardia; pallor; tremor; nausea; diarrhea;	Nocturnal anaphylaxis	None	No	n.a.	Yes;<br><br>Diarrhea, abdominal cramps	No	n.a.	No additional information
**P14**	39	f	No atopy; contact urticaria to horse saliva;	Angioedema, urticaria; tingling of the tongue; crescendo reaction: additional gastrointestinal symptoms like cramps;	3-4 hrs	Exercise; alcohol; stress; bronchial infection	Yes	none	No	?	n.a.	Reactions to bovine collagen;<br><br>Intolerance to Lab

**P15**	47	f	Suspected inhalant allergy in spring; suspected wheat hypersensitivity; Known celiac disease.	Consumption of red meat (beef, pork) induces OAS after 30 min; after 2 hrs: abdominal pain, flatulence, diarrhea; reaction to Kreon (contains gelatin) and milk (the latter with the addition of skin papules; Once a reaction to poultry sausage which contained bovine proteins/gelatin	30 min (OAS) GI symptoms after 2 hrs	Hereditary α-trypta-semia	Yes	none	Papules on skin	Yes, in medication (Kreon®)	n.a.	Tick bite with suspected borreliosis
**P16**	48	f	No atopy; large local reaction on the leg after wasp sting	Nocturnal pruritus, urticaria; After roast rabbit: generalized urticaria, flush, pruritus; dizziness, nausea, vomitus, diarrhea; Milk induces diarrhea.	3 hrs	none	Yes	none	Yes: diarrhea	No	n.a.	Tick bites with subsequent nocturnal reactions; reactions to a poultry sausage containing cheese
**P17**	36	f	Inhalant allergy to grass pollen; Pollen-associated food allergy	Recall urticaria; dizziness, retrosternal pressure; dyspoea; Milk and dairy increase gastrointestinal symptoms	6-7 hrs	none	Yes	Swelling at the tick bite site after meat consump-tion and allergic reaction to meat	Yes, milk and dairy increase gastrointest-inal symptoms	No	n.a.	Tick had to be removed surgically; weeks later, first reaction to red meat with recalled urticaria
**P18**	52	m	Inhalant allergy (tree pollen); food intolerance (orange, pear, glutamate)	Worsening of eczema (prurigoform/atopic?); flatulence	6-10 hrs	none	yes	none	No	No	n.a.	Tick bites before symptom development
**P19**	42	f	Inhalant allergy (tree pollen; herbal pollen); pineapple (OAS)	Nocturnal gastrointestinal pain; nausea, generalized urticaria, eventually dyspnea	Several hours	Alcohol. Probably NSAID during one event	Yes	none	No	No	n.a.	No additional information

**P20**	51	m	Inhalant allergy (tree pollen, cat dander); Food allergy	Abdominal cramps; diarrhea; Dose dependency.	2-3 hrs	none	Yes	none	No	No	n.a.	Borrelia-IgG+
**P21**	59	m	Inhalant allergy to HDM	Urticaria	3 hrs	Alcohol at the first event	Yes	none	No	No	n.a.	Tick bites; Borreliosis; recipient of prosthetic heart valves 30 yrs ago
**P22**	59	f	Penicillin allergy, contact allergy cobalt chloride	Recurrent severe urticaria and pruritus, crescendo: additional dyspnea, angioedema, increase in gastrointestinal activity, diarrhea. Reaction to gelatin in gum bears	6 hrs	none	Yes	none	No	Reaction to consumption of gummy bears	n.a.	Reaction to poultry sausages (contained bovine material)
**P23**	33	m	Inhalant allergy (grass pollen); asthma	Recurrent swelling of the inguinal lymph nodes, subsequently followed by generalized urticaria, flush, nausea and pruritus, often at night, gastro-oedema. Dose dependency of the meat meals.	4-5 hrs	none	Yes	none	Yes; Nausea 3-5hrs after dairy (yoghurt)	No	n.a.	Many tick bites
**P24**	45	f	Inhalant allergy to HDM and cat in childhood. Food allergy, Protein-contact dermatitis (fish), EoE, Asthma. Sensitization to some plant foods and LTPs.	Gastrointestinal symptoms dominant: butter milk: sneezing, gastrointestinal cramps, diarrhea; itchy throat; swelling of the throat; dyspnea; the feeling as if the esophagus becomes tight. After cow's milk abdominal cramps and diarrhea. OAS	30 min	Stress, exercise, coffee	Yes	none	Yes, Abdominal cramps and diarrhea	No; but prospective avoidance	n.a.	Symptoms to different foods with no regularity. After avoidance of red meat improvement; professional cook
**P25**	61	m	Inhalant allergy, suspected α-Gal-allergy,	Urticaria, feeling of heat, (sausages, mixed meat (pork and beef))	15 min to 3 hrs	Emotional stress	No	n.a.	No	No	n.a.	No additional information
**P26**	69	m	Inhalant allergy to grass and birch pollen;	Pruritus, urticaria, dyspnea; drop of blood pressure (after a wheat roll)	No delay	exercise	Yes	none	No	No	n.a.	Consumption of red meat (beef, pork, common deer)

			pollen-associated food allergy. Wheat: WDEIA, **no AGS**									
**P27**	21	m	No atopy. Sensitization to gluten > banana>kiwi with unclear relevance	EoE and heartburn	Not certain in the case the EoE develops after meat ingestion	none	yes	none	No	No	n.a.	Pets: cats Eats meat regularly; meat consumption does not induce heartburn
**P28**	62	m	Inhalant allergy to tree pollen	Pruritus, angioedema, urticaria; palmo-plantar pruritus; erythematous hands	Several hours	none	Yes	none	No	Not sure	n.a.	Tick bites 12 years ago; suspicion of reaction to bovine collagen (in poultry sausages; calf rennet), lab
**P29**	62	f	No atopy. Rash and generalized pruritus after consumption of some antibiotics	Feeling of fullness, flatulence, abdominal cramps, diarrhea; nausea	2-3 h	none	No	n.a.	No; Tolerance instead	Yes, GI-symptoms	n.a.	No additional information
**P30**	37	m	No atopic diseases. Milk allergy	Recurrent pruritus and rash, itchy throat and irritative cough with subsequent dyspnea, nausea, diarrhea, circulation problems; after consumption of a glass of milk: abdominal cramps and diarrhea	3-4 hrs GI: 30-60 min	none	Yes	none	Yes: abdominal cramps, diarrhea	No	n.a.	Repeated tick bites
**P31**	57	f	No atopy. Food intolerance (fruits, wheat); meat tolerance, but intolerance to milk	Heartburn and diarrhea	30-60 min	none	No	n.a.	Yes, but does-dependence	No	n.a.	No additional information
**P32**	43	f	Inhalant allergy (pollen, animal dander), possibly HDM	Nausea nearly to vomitus, dizziness	7 hrs	Menstruation stress, NSAIDs possible	Yes	With accompanyi ng skin reaction	No	No	n.a.	Recurrent tick bites; no borreliosis

**P33**	67	f	Inhalant allergy (pollen, HDM)<br><br>Pollen-associated food allergy; a-Gal syndrome; tolerance to milk	Prickling and subsequent swelling of hands and feet; dizziness, angioedema; generalized urticaria and itching, tachycardia, diarrhea; flush; dyspnea	7 hrs	alcohol	Yes	Persistent reaction	No;<br><br>Tolerance instead	Skin sensitization, avoids gelatin	n.a.	Tick bite in 2021 several months before reaction. Increase in anti-a-Gal-IgE after new tick bite in 2024

Table S2. ImmunoCAP analysis of patient sera. Results for total IgE, tryptase, and Bos d TG / total IgE ratio. Grey background indicates patients with negative serum test for IgE against Bos d TG (α-Gal analyte) in ImmunoCAP (see Table 1).

ID	total IgE [kU/L]	tryptase [µg/ml]	Bos d TG: total IgE ratio
P01	119	3.2	0
P02	294	2.9	0
P03	137	3.6	0
P04	134	4.7	0.04
P05	1773	5.4	0
P06	573	3.3	0.17
P07	122	4.3	0
P08	188	6.9	0.15
P09	723	5.5	0
P10	121	7.8	0.04
P11	594	3.1	0.06
P12	1517	4.2	0.01
P13	401	3.9	0
P14	60	5.2	0.51
P15	22	10.2	0
P16	261	4.4	0.08
P17	23	6.0	0.36
P18	37	6.9	0
P19	370	4.0	0.11
P20	807	8.7	0.03
P21	878	3.5	0.07
P22	95	4.8	0.34
P23	67	4.1	0.09
P24	384	6.0	0.03
P25	741	4.8	0
P26	157	6.1	0.01
P27	>2500	4.4	0
P28	126	4.6	0
P29	110	9.0	0
P30	567	3.0	>0.18
P31	86	5.0	0.02
P32	20	5.5	0.11
P33	47	6.3	0.12

Table S3. Grouping of ICRIP reaction patterns. Positive signals are indicated by "+". Sera with negative test for IgE against Bos d TG (α-Gal) in ImmunoCAP are written with a grey background (see Table 1).

"Full α-Gal-analyte pattern CTX/HSA-α-Gal$_3$/Bos d TG" (see Figure 2).

ID	CTX	IFX	HSA	HSA-α-Gal$_3$	Bos d TG	Bos d 6
P06	+	-	-	+	+	-
P08	+	-	-	+	+	-
P11	+	-	-	+	+	-
P12	+	-	-	+	+	-
P14	+	-	-	+	+	-
P16	+	-	-	+	+	-
P17	+	-	-	+	+	-
P19	+	-	-	+	+	-
P21	+	-	-	+	+	-
P22	+	-	-	+	+	-
P23	+	-	-	+	+	-
P33	+	-	-	+	+	-

CTX only.

ID	CTX	IFX	HSA	HSA-α-Gal$_3$	Bos d TG	Bos d 6
P13	+	-	-	-	-	-
P20	+	-	-	-	-	-
P24	+	-	-	-	-	-
P26	+	-	-	-	-	-
P28	+	-	-	-	-	-
P32	+	-	-	-	-	-

TG only.

ID	CTX	IFX	HSA	HSA-α-Gal$_3$	Bos d TG	Bos d 6
P01	-	-	-	-	+	n.d.
P02	-	-	-	-	+	-
P03	-	-	-	-	+	-
P18	-	-	-	-	+	-
P29	-	-	-	-	+	-

Mixed patterns.

ID	CTX	IFX	HSA	HSA-α-Gal_3	Bos d TG	Bos d 6
P04	**+**	-	-	-+	**-**	-
P09	**+**	-	-	-	**+**	**+**
P10	**+**	+	-	-	**-**	-
P27	**+**	**-**	-	-	+	+
P30	**+**	**+**	-	**+**	**+**	-
P31	**+**	-	-	**-**	+	-

α-Gal - IgE-negative.

ID	CTX	IFX	HSA	HSA- α-Gal_3	Bos d TG	Bos d 6 / BSA
P05	-	-	-	-	-	-
P07	-	-	-	-	-	-
P15	-	-	-	-	-	-
P25	-	-	-	-	-	**+**
